# Lack of association between bovine leukemia virus and breast cancer in Chinese patients

**DOI:** 10.1186/s13058-016-0763-8

**Published:** 2016-10-10

**Authors:** Rong Zhang, Jingting Jiang, Weihong Sun, Jilei Zhang, Ke Huang, Xuewen Gu, Yi Yang, Xiulong Xu, Yufang Shi, Chengming Wang

**Affiliations:** 1 Jiangsu Co-innovation Center for Prevention and Control of Important Animal Infectious Diseases and Zoonoses, Yangzhou University College of Animal Science and Technology, Yangzhou, China; 2 Department of Tumor Biological Treatment, The Third Affiliated Hospital of Soochow University, Changzhou, China; 3 Institute of health Sciences, Shanghai Institutes for Biological Sciences (SIBS), Chinese Academy of Sciences (CAS) & Shanghai Jiao Tong University School of Medicine, Shanghai, China; 4 Yangzhou University College of Medicine, Yangzhou, Jiangsu China; 5 The First Affiliated Hospital of Soochow University, Institutes for Translational Medicine, Soochow University, Suzhou, China; 6 Department of Pathobiology, College of Veterinary Medicine, Auburn University, Auburn, AL USA

Since Francis Peyton Rous’s landmark study of identifying avian oncogenic virus, seven viruses have been found to cause 10–15 % of human cancers worldwide [1]. Recent findings by Buehring’s group have suggested that another virus, bovine leukemia virus (BLV) – which is highly prevalent in cows worldwide [2] – might be linked to human breast cancer [3–5]. In a study of 218 women from four states in the USA, Buehring’s group showed that the BLV positivity in women with breast cancer (67/114) is significantly higher than that in women without breast cancer (30/104) [4].

In this study, we conducted seven PCRs according to Buehring’s group [3, 4] and one published real-time PCR to detect BLV in breast cancer tissue (*n* = 91) and blood (*n* *=* 160) from Chinese women with breast cancer from four tissue banks in Anhui, Jiangsu, and Shanghai, in blood from women without breast cancer in Anhui (*n* = 100), and in blood from cows in Anhui, Beijing, Heilongjiang, and Jiangsu (*n* = 150).

PCR analysis revealed that BLV was positive in 50.0 % of the cattle blood samples but was undetectable in the blood from women with or without breast cancer. ELISA revealed that 47.3 % of the cattle blood samples were BLV antibody positive, whereas none of the blood samples from women with or without breast cancer were BLV antibody positive. Although eight published PCRs in our study could readily detect BLV in the blood samples of herds with a high percentage of positivity, these PCRs did not show positive for BLV in the samples of either breast tumor tissues or blood from Chinese women with breast cancer. Similarly, the commercial ELISA kit used in our study could readily detect BLV-specific antibodies in all cows which were also positive for BLV by PCR, but failed to detect reactive antibodies in Chinese women with or without breast cancer. Although information on milk consumption in women participating in our study was not available, our prior study showed a high infection rate of BLV in cows from three provinces where our human samples were collected [2].

The strong association between BLV and human breast cancer would raise serious public health concerns worldwide. Here we provide unambiguous data showing no evidence of BLV in Chinese women with or without breast cancer. Further investigations are warranted to explore the association of BLV with breast cancer in patients with different genetic backgrounds and from different countries as well as their exposure to milk and the levels of infection in dairy herds.
